# Evaluation of Nosocomial Infection in Patients at hematology-oncology ward of Dr. Sheikh children’s hospital

**Published:** 2015-12-10

**Authors:** A Ghassemi, H Farhangi, Z Badiee, A Banihashem, MR Mosaddegh

**Affiliations:** 1 Associate professor of pediatric Hematology& Oncology & stem cell transplantation, Mashhad University of medical sciences, Mashhad, Iran.; 2 Assistant professor of pediatric Hematology& Oncology, Mashhad University of medical sciences, Mashhad, Iran.; 3 Associate professor of pediatric Hematology& Oncology, Mashhad University of medical sciences, Mashhad, Iran.; 4 Professor of pediatric Hematology& Oncology, Mashhad University of medical sciences, Mashhad, Iran.; 5 Head nurse of infectious control at Dr Sheikh children’s hospital, Mashhad University of medical sciences, Mashhad, Iran.

**Keywords:** Nosocomial Infection, Urinary, Respiratory, Blood Stream Infection

## Abstract

**Background:**

Infections in critical care unit are high, and they are serious hospital problems. Infections acquired during the hospital stay are generally called nosocomial infections, initially known as infections arising after 48 h of hospital admission. The mostfrequent nosocomial infections (urinary, respiratory, gastroenteritis and blood stream infection) were common in patients at hospital.The aim was to study, the current status of nosocomial infection, rate of infection among hospitalized children at hematology-oncology ward of Dr. Sheikh children’s hospital, Mashhad, Iran.

**Materials and Methods:**

Data were collected from 200 patient's records presented with symptoms of nosocomial infection at hematology-oncology ward of Dr. Sheikh children’s hospital from March 2014 to September 2014. Descriptive statistics using percentage was calculated.

**Results:**

Incidence of nosocomial infections inpatients athematology-oncology ward was 31% (62/200). Of which 69.35% (43/62) blood stream infection being the most frequent; followed by 30.64% (19/62) was urinary tract infection (UTI), and the most common blood culture isolate was been Staphylococcus epidermidis 18 (41.86%), andour study showed that large numbers ofnosocomial UTIs causing by Gram‑negative bacteria.

**Conclusion:**

This study showed blood stream infection and UTI are the common nosocomial infections among patients athematology-oncology ward. Early recognition of infections and short term use of invasive devices along with proper infection control procedures can significantly decrease the incidence of nosocomial infections in patients.

## Introduction

A global problem in every hospital around the world is Nosocomial infections that are also named healthcare-associated infections (HAI) (1). These kinds of infections arise 48 hours after hospital admission. Nosocomial infections are major cause of mortality and morbidity among hospitalized patients (2). These infections may be classified as primary when no focus identified (3) or secondarily 

when localized infection at a specific body site identified. They are manifested with signs of septic and sepsis shock (4). The most frequent nosocomial infections, namely urinary, respiratory, gastroenteritis, and blood stream infection are common in hospitalized patients (5).The risk factors of nosocomial infections include: intubation, poor health status, diabetes mellitus, surgical drains, persistent sounding, and lack of using gloves (6).

The most common type of nosocomial infections is urinary tract infections (UTIs). Nosocomial UTIs can provide important information for decision making in terms of individual hospitalization regarding potential outbreaks, antimicrobial resistance, local trends, and unusual pathogens in the etiology of infections (7).

Bloodstream infections(BSI), as a complication of critical illness, cause severe sepsis, septic shock, and multisystem organ dysfunction (3).More recent studies demonstrated a reduction of BSI in patients from 1.09 to 0.89/100 and from 2.04 to 0.75/100 after implementation of specific control measures (8,9).The main stay therapy for patients with bacteremia is antimicrobial therapy especially initial antimicrobial therapy; although, empirical has an important impact upon the survival of patients with BSI with the optimal management of its consequences as well as surgical treatment of an infection site when appropriate (1).

The magnitude of this problem in a developing country is even more serious since there is no available established statistics. Even though it is not possible to eradicate the nosocomial, many of them can be prevented by proper control measures. This study aimed at estimating the incidence rate of nosocomial infections between hospitalized children at hematology-oncology ward of Dr. Sheikh children’s hospital.

## Materials and Methods

**Patients**

The current study was a sectional analytical study. The data for the current study came from hospitalized children, younger than 18 years old, with symptoms of nosocomial infection in their records and through analysis of their infections. Totally, the data of this study included 200 patients records hospitalized at hematology-oncology ward of Dr. Sheikh children’s hospital from March 2014 to September 2014. All of patients admitted in hematology-oncology ward included and every patient dead with other cause of infectious exclude from our study.

**Sample collection and processing**

 In order to culture blood for determining BSI, 5ml of blood was inoculated into ready prepared culture vials and incubated into the automated system. The same system was applied for identification of microorganisms.

The urine samples were collected by midstream clean catch method and were sent to the hospital’s microbiology laboratory and then were cultured. If microbial growth occurred, differential cultures and tests were performed to identify different bacterial strains. Bacterial species were identified through different microbiologic tests.

**Statistical analysis**

Statistical analysis was done through SPSS software version 19 and p-value less than 0.05 was considered significant.

## Results

Total of 200 children, who were at hematology-oncology ward of Dr. Sheikh children’s hospital from March 2014 to September 2014, were enrolled in the survey. Based on the exclusion and inclusion criteria, 62 patients were positive for nosocomial infections about 31%. The blood culture positivity rate in our hematology-oncology ward was estimated to be 69.35% (43/62). In terms of urinary culture, about 30.64% (19/62) were positive for urinary tract infections (UTI). No gastroenteritis infection was reported in patients (Table I). Therefore, bloodstream infection was recognized as the most frequent nosocomial infections among children at hematology-oncology ward. Total of 43 samples were positive out of 62 blood cultures that were collected from the study group who were positive for nosocomial infections. The blood culture positivity rate in our internal section was estimated to be 69.35%. Microorganisms were isolated from infections included the phrase of Staphylococcus epidermidis 18 (41.86%), Pseudomonas aeruginosa 12 (22.90%), Enterococcus 5 (11.62%), Ecoli 5 (11.62%), Acinetobacter2 (4.65%), and Klebsiella 1(2.32%) (Table II).

Out of 62 urine samples, 19 (30.64%) had positive culture for UTIs. Among nosocomial infections, microorganisms which were isolated from infections included: the phrase of E. coli 12 (63.15%), Enterococcusand Staphylococcus epidermidis 2 (10.52%), Streptococcus pneumoniae, Proteus mirabilis, and Klebsiella 1 (5.2%) (Table III).

**Figure 1:**
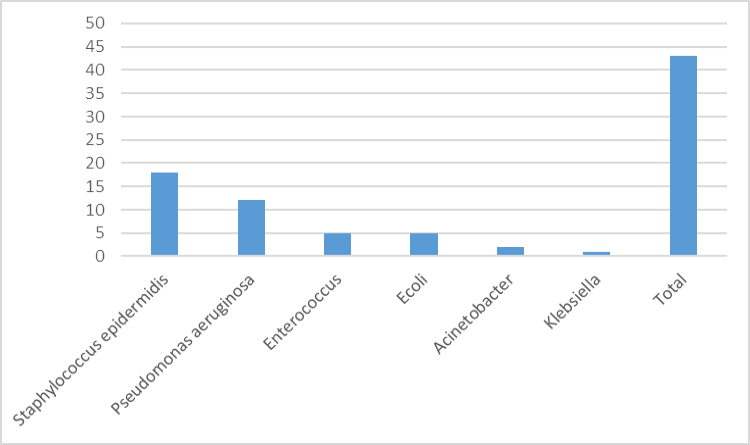
Distribution of the various microorganisms from blood culture from hematology-oncology ward patients

**Figure 2:**
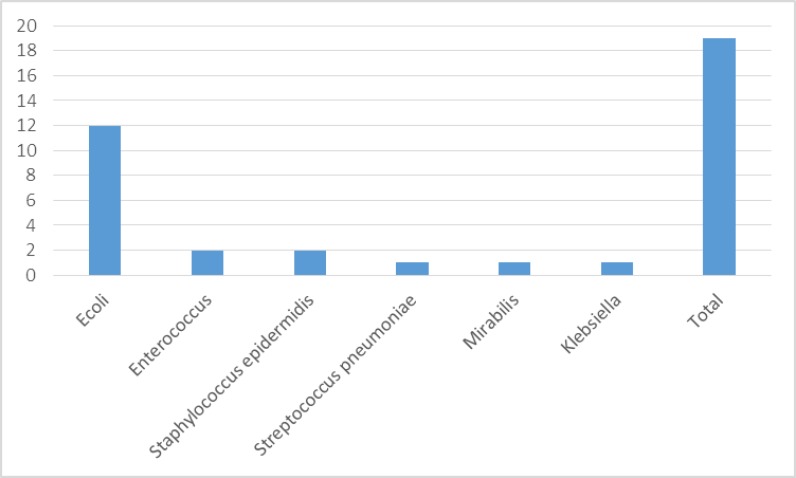
Distribution of the various microorganisms from urine culture from hematology-oncology ward patients

**Table I:** Distribution of nosocomial infections among nosocomial positive patients

**Nosocomial infection**	**Number of patients**	**Percentage**
**Urinary tract infection**	19	30.6
**Blood stream infection**	43	69.4
**Gastrointestinal infection**	0	0
**Total**	62	100

**Table II:** Microorganisms isolated in blood cultures

Kind of Bacteria	Abundance	Percentage
Staphylococcus epidermidis	18	41.86
Pseudomonas aeruginosa	12	27.93
Enterococcus	5	11.62
Ecoli	5	11.62
Acinetobacter	2	4.65
Klebsiella	1	2.32
Total	43	100

**TableIII:** Microorganisms isolated in urinary cultures

Kind of Bacteria	Abundance	Percentage
E. coli	12	63.15
Enterococcus	2	10.52
Staphylococcus epidermidis	2	10.52
Streptococcus pneumoniae	1	5.3
Mirabilis	1	5.3
Klebsiella	1	5.3
Total	19	100

## Discussion

In this study was to evaluate the incidence of nosocomial infections inhematology-oncology ward at Dr. Sheikh children’s hospital, determining risk factorsis identified as ways to control infections is provided in a hospital. In present study first we evaluated the nosocomial infections among children at hematology-oncology ward;the result show that rate of nosocomial infections was 31% (62/200),Nosocomial infections in different areas at the same studies were been different for example; Mythriet alreported that in 130 patients in hospital Medical Intensive Care Unit (MICU) twenty-three out of 130 (23/130-17.7%) admitted to the MICU suffered from nosocomial infection [5];Signorelli et al. demonstrated at children’s hospital in Italyof 487 children enrolled in their survey, 25 (5.1%) had nosocomial infection (11); Ahoyoet al. reported the prevalence of nosocomial infection between hospitals in Benin was been 19.1% (12); Richards et al. described the 68% nosocomial infections at intensive care units (ICUs) in United Sates (13). According to the results of this study seem the rates of nosocomial infectionhave been similar to another studies. 

 The general distribution pattern of the nosocomial infections in our study showed bloodstream infection(BSI)to be the most common nosocomial infections between children at hematology-oncology ward 69.35% and the most common blood culture isolate was been Staphylococcus epidermidis 18 (41.86%) and Pseudomonas aeruginosa 12(22.90%) (Figure 1). Some other studies done by Mukherjee et al., 61% as well as from other studies done byJaponiet al., 67.7% andKarlowskyet al., 42%, also observed as the commonest BSI (14, 15). Koupetoriet al. reported the rate of BSI in patients at emergency 68% and at general ward 42.9% (16), Wattalet al. for blood culture positivity was estimated 67.5% at care unit patients, which Gram negative bacilli and Gram positive cocci were isolated in 49% and 33% cases, respectively (17).Valles et al. reviewed more than five recent BSIfrom different parts of the world observed,Gram positive cocci as the commonest microorganism (20–30%) causing BSIs among ICU patients (18). The findings of the current study suggest that theBSIto be the most common nosocomial infections at hematology-oncology ward. Although Gram-negative bacteria and Gram positive cocci were the commonest microorganism causing BSIs among patients at hematology-oncology ward. The result reinforces the need to implement specific control measures to decrease the spread of microorganism’s infection in these patients.

UTI is another the most common bacterial infections in children at hematology-oncology ward and the Gram-negative bacteria are the most common etiological agents involved. We demonstrated the rate of UTI was 30.64% and that large numbers of Gram‑negative bacteria causing nosocomial UTIs, especially E. coli (63.15%) (Figure2).AngèleAhoyoet al. reported the UTIs (48.2%) The most frequent infections of Benin hospitals and 67.6% ofnosocomial UTIs due to E. coli (12).Mythriet al.showed nosocomial UTIs (34.8%) to be the most commonnosocomial infectionspatients in ICU (5). Soltaniet al. demonstrated at their studies in 213 urine samples had growth of Gram‑negative organism. E. coli was the most frequently isolated organism (61%), followed by K. pneumonia (17.8%), P. aeruginosa (12.2%), and A. baumannii (4.2%) (19). Several other studies in Iran (20,21) and other countries (22-24) reported the similar results for E. coli as the most frequentpathogens causing nosocomial UTI.Interestingly, in a prospective study conducted in the Calgary

Health Region, 28% of E.coli infections were healthcare-acquired (25). Our study showed that large numbers of nosocomial UTIscausing byGram‑negative bacteria and these data indicate the risk that inthe near future antibiotic-resistant Gram-negativebacteria might represent a severe problemfor children at hospital.

## Conclusion

This study showed BSIandUTI are the common nosocomial infections among patients athematology-oncology ward,the most common blood culture isolate was been Staphylococcus epidermidis(Gram positive cocci) andthe large numbers ofnosocomial UTIscausing byE.coli (Gramnegative bacteria). Early recognition of infections and short term use of invasive devices along with proper infection control procedures can significantly decrease the incidence of nosocomial infections in patient, therefore we can decrease amount of mortality and morbidity among children hospitalized at hematology-oncology ward.
